# Rare strangulated intenal hernia following extraperitoneal colostomy for rectal cancer operation: A case report

**DOI:** 10.1016/j.ijscr.2024.109911

**Published:** 2024-06-15

**Authors:** Yosuke Kotohata, Mizunori Yaegashi, Noriyuki Sasaki, Akira Sasaki

**Affiliations:** Department of Surgery, Iwate Medical University School of Medicine, Shiwa, Japan

**Keywords:** Rectal cancer, Extraperitoneal route, Abdominoperineal resection, Internal hernia, Case report

## Abstract

**Introduction:**

Few cases of intestinal obstruction after colostomy are caused by internal hernia. Some institutions perform stomas through the extraperitoneal route because some patients experience an internal hernia outside the stoma performed through the intraperitoneal route.

**Presentation of case:**

A 72-year-old woman presented with a history of laparoscopic abdominoperineal resection (APR). A sigmoid colostomy was performed via the extraperitoneal route during APR. One month after APR, the patient presented to the emergency department of our hospital with abdominal pain and vomiting. Computed tomography revealed that the small intestine had passed through the extraperitoneal tunnel, resulting in strangulated intestinal obstruction, and emergency laparotomy was performed. During surgery, the ileum passed behind the elevated sigmoid colon in a caudal-to-cranial direction and formed an unusual closed loop. The strangulated part of the small intestine showed ischemic change; however, the intestine quickly normalized soon after strangulation was released, and the operation was completed without resection of the intestine.

**Discussion:**

The major cause of intestinal obstruction after colostomy is intraperitoneal adhesion. Looseness of the elevated sigmoid colon can cause internal hernia, if under pneumoperitoneum, when a colostomy is created through the extraperitoneal route in laparoscopic APR. Furthermore, the patient had lost more than 5 kg of body weight after the surgery, which may have led to the looseness of the elevated sigmoid colon.

**Conclusion:**

Releasing the pneumoperitoneum during the elevation of the sigmoid colon is necessary to prevent internal hernia, even with a colostomy performed through the extraperitoneal route.^1^

## Highlights

• Some institutions perform stomas through the extraperitoneal route because some patients experience an internal hernia.
• We experienced an atypical internal hernia which entered from the caudal side after extraperitoneal colostomy.
• If pelvic cavity is closed during the first operation, the ileum can enter from the caudal side of the elevated colon and form an internal hernia.
• Some ideas such as lowering pneumoperitoneal pressure before raising the colostomy can be solutions to prevent the internal hernia.

## 1 Introduction

When a permanent colostomy is created laparoscopically, some institutions use the extraperitoneal route because some patients experience an internal hernia from the head to the outside of the stomas created through the intraperitoneal route [1]. In this case, we experienced an unusual internal hernia from the caudal lateral side of the elevated sigmoid colon, even though we created a stoma through the extraperitoneal route during laparoscopic abdominoperineal resection (APR). This case report was prepared according to the SCARE Criteria [2].

## 2 Presentation of case

**Fig. 1:**
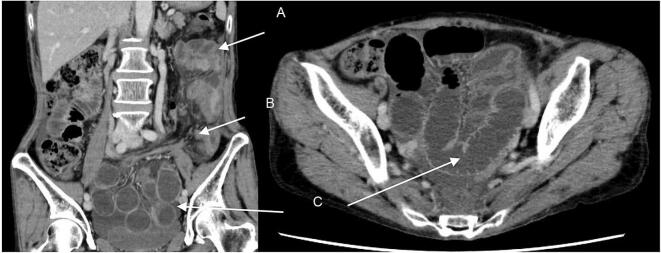
Computed tomography reveals an internal hernia causing strangulated intestinal obstruction due to the small intestine (A) entering between the elevated colon (B) and abdominal wall. The small intestine on the cranial side of the strangulation is dilated throughout (C).

A 72-year-old woman was diagnosed with lower rectal cancer. The patient underwent laparoscopic APR and resection of the posterior vaginal wall at our hospital. Although she received neoadjuvant chemotherapy with the CAPEOX regimen, she received only two courses due to adverse events. We created a stoma through the extraperitoneal route. The pelvic cavity was closed with continuous peritoneal sutures, and AdSpray® (Terumo) was used as a spray-type antiadhesive agent. She needed long time for her to learn how to replace her stoma pouch. Therefore, she was discharged from hospital 19 days after surgery with no complications. Although, the patient presented to the emergency department with abdominal pain and vomiting 38 days after the surgery. The patient had a history of duodenal ulcers. When she came to the hospital, the patient had no fever, but had abdominal pain in the left lower quadrant area. On admission, the laboratory findings revealed a high white cell count (17,230/μL), and the C-reactive protein level was 0.31 mg/dL. Abdominal radiography revealed the air fluid levels and dilation of the small intestine, suggesting intestinal obstruction. Computed tomography revealed that the small intestine passed through the space between the colostomy created through the extraperitoneal route and the abdominal wall, resulting in strangulated intestinal obstruction due to internal hernia (Fig. 1). Based on these findings, the patient was diagnosed with a strangulated intestinal obstruction due to the ileum entering the extraperitoneal tunnel and forming an internal hernia, and an emergency laparotomy was performed.

**Fig. 2:**
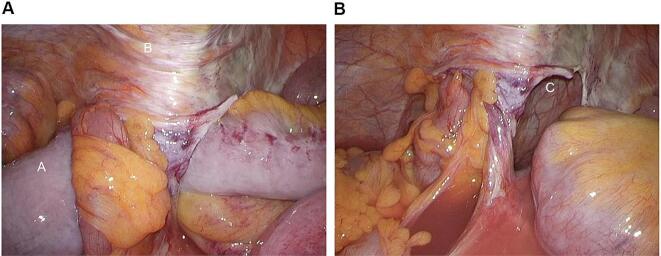
a: The small intestine formed a closed loop (A) behind the elevated sigmoid colon (B). b: When the strangulated small intestine was withdrawn, an extraperitoneal tunnel was observed between the sigmoid colon and abdominal wall.

During the surgery, a part of the small intestine forming a closed loop was observed on the cranial side of the elevated sigmoid colon (Fig. 2a). The small intestine entered an area 50–60 cm from the end of the ileum. We attempted to pull out the strangulated ileum from the caudal side; however, this was difficult because of the sclerotic peritoneum. Therefore, we made an incision in the peritoneum to pull out the ileum and release the strangulation (Fig. 2b). The strangulated part of the small intestine was highly edematous and showed ischemic change; however, the ischemic intestine normalized approximately 20 min after strangulation was released, and the operation was completed without the resection of the intestine. We made a peritoneal incision to prevent the recurrence of internal hernia and placed a drain in the pelvis. The operative time was 60 min, and the blood loss was 1 ml. We allowed walking and drinking water on the day after surgery. The patient began kept Npo for 3 days after surgery and was discharged 11 days after surgery with no complications. No recurrence of internal herniation was observed during follow-up.

## 3 Discussion

A colostomy is often created via the intraperitoneal route during laparoscopic Hartman's surgery or APR. Major complications after colostomy include parastomal hernia, stoma stenosis, and intestinal obstruction, which occur in 10 %–70 % of patients [3,4]. The main cause of intestinal obstruction is postoperative intra-abdominal adhesion, and few cases are caused by internal hernia after colorectal surgery [5,6]. When a single-hole colostomy is performed during APR or Hartmann surgery, there are cases of internal hernia in the lateral space of the stoma created through the intraperitoneal route [1,7]. To prevent internal hernias after colostomy, Goligher performed stoma construction via the extraperitoneal route to avoid the occurrence of internal hernias [8]. In other case, Yasukawa et al. suggested creating an extraperitoneal colostomy and closing the cranial orifice [1].

**Fig. 3:**
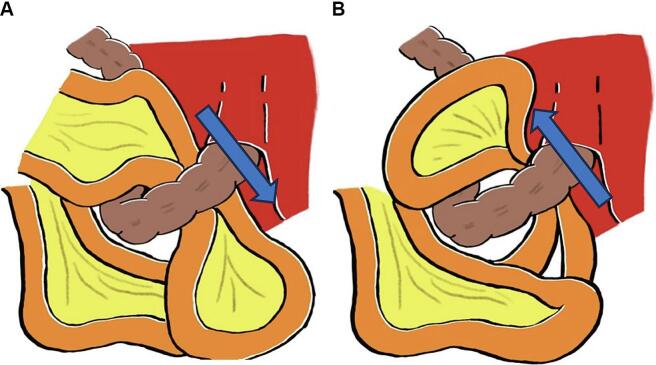
a: In past reports, the small intestine passed through in a cranial-to-caudal direction. b: In this case, the small intestine passed through in a caudal-to-cranial direction.

**Fig. 4:**
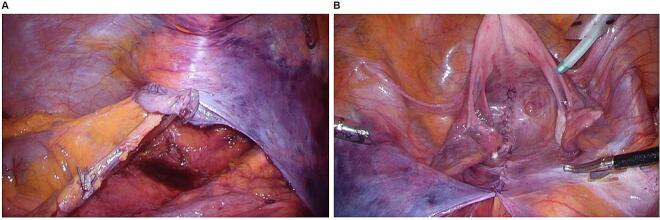
We elevated the sigmoid colon via the extraperitoneal route (a) and closed the pelvic cavity during the first operation (b).

The laparoscopic extraperitoneal colostomy technique was developed to avoid the occurrence of parastomal hernia. To the best of our knowledge, two cases [9,10] have been reported, in which the small intestine entered between the extraperitoneal colostomy and the abdominal wall, resulting in internal hernia (Table 1). All of these cases were performed via laparoscopic APR, which may have made adhesions less likely to occur. In one of the two cases, blood flow did not improve, and the small intestine was partially resected. Furthermore, in previous reports, the small intestine passed through in a cranial-to-caudal direction (Fig. 3a), whereas, in our case, it passed through in a caudal-to-cranial direction (Fig. 3b). We created a colostomy via the extraperitoneal route (Fig. 4a) and closed the pelvic cavity during the first operation (Fig. 4b). In addition, the use of a spray-type antiadhesive agent and laparoscopic approach reduced adhesion and allowed more freedom of the small intestine, so that ileum may have resulted in the occurrence of internal hernia through in a caudal-to-cranial direction. In this case, closing the space between the extraperitoneal colostomy and the abdominal wall during the first operation, or avoid too much dissection of peritoneum during extraperitoneal colostomy, may also prevent internal hernia. As another precaution, raising the colostomy outside the body after lowering the pneumoperitoneal pressure at the time of colostomy is considered necessary because of the reduction in the space outside of extraperitoneal colostomy.

**Table 1:** Reported cases of internal hernia after colostomy through the extraperitoneal route.

Study	Year	Age	Sex	Type of surgery	Direction	Strangulated intestine	Resection of the intestinal
Yokota et al. [9]	2013	70	Male	Laparoscopic APR	Cranial-to-caudal	Ileum	Yes
Yokoyama et al. [10]	2014	56	Female	Laparoscopic APR	Cranial-to-caudal	Ileum	No
Our case	2023	72	Female	Laparoscopic APR	Caudal-to-cranial	Ileum	No

## 4 Conclusion

We report a case of strangulated intestinal obstruction with an extraperitoneal tunnel as the portal of the hernia after an extraperitoneal colostomy. We suggest closing the extraperitoneal tunnel and releasing the pneumoperitoneum during the elevation of the intestine to prevent internal hernia when we create a colostomy through the extraperitoneal route.

## Consent

Written informed consent was obtained from the patient for publication of this case report and the accompanying images. A copy of the written consent is available for review by the Editor-in-Chief of this journal on request.

## Ethical approval

The anonymised presentation of case report does not require a separate approvement by the ethics committee in our institute.

## Funding

The authors didn't use any sources of funding, and this study has no sponsors.

## Author contribution

Yosuke Kotohata and Mizunori Yaegashi conceived the case presentation and drafted the manuscript.

Mizunori Yaegashi, Noriyuki Sasaki and Akira Sasaki have read and approved the final manuscript.

## Guarantor

Mizunori Yaegashi.

## Research registration number

None.

## Declaration of competing interest

There are no conflicts of interest for authors in this study.
